# DEMENTIA LITERACY ENHANCEMENT DESIGN AND ITS OUTCOMES IN TAIWAN

**DOI:** 10.1093/geroni/igae098.2352

**Published:** 2024-12-31

**Authors:** Tsuann Kuo, An Chi Lai

**Affiliations:** Chung Shan Medical University, Taichung, Taichung, Taiwan (Republic of China); National Yunlin University of Science and Technology, Yunlin, Yunlin, Taiwan (Republic of China)

## Abstract

**Background:**

The increasing number of people diagnosed with Dementia requires the public to become more knowledgeable about dementia and to act more dementia friendly.

**Purpose:**

This paper was to evaluate the effectiveness of a dementia education effort. The purpose was to increase dementia literacy and find out what aspects the public changed the most on dementia.

**Methodology:**

A pre- and post-test was administered to 1237 individuals who attended the dementia education sessions. Each session lasted one hour and 38 sessions were administered within three months at various locations including community care sites, churches, cultural centers, stores, and schools. A pre- and post- test on dementia literacy and attitudes were administered and the response rate was 68%. The majority were females (80%), 60 to 70 years old (25.94%), with a university degree (53.71%), and retired (27.66%).

**Results:**

One-way ANOVA analyses revealed significant associations between participants’ age group, educational level, and the difference in the pre-post test (p< 0.01). The results suggest that the educational intervention was effective in enhancing dementia literacy, but the degree of improvement varied across different demographic groups and locations.

**Discussion and conclusion:**

The dementia education sessions effectively improved dementia literacy, with variations observed across age and educational groups. Tailoring educational content and delivery methods to cater to diverse demographic backgrounds could further enhance the effectiveness of such initiatives in promoting dementia awareness and fostering a more inclusive society. Future research should explore specific strategies to optimize educational programs for different target populations, ultimately contributing to a more dementia-friendly community.

